# Metabolic Profiling and In Vitro Assessment of the Biological Activities of the Ethyl Acetate Extract of *Penicillium chrysogenum* “Endozoic of *Cliona* sp. Marine Sponge” from the Red Sea (Egypt)

**DOI:** 10.3390/md20050326

**Published:** 2022-05-15

**Authors:** Muneera S. M. Al-Saleem, Wafaa H. B. Hassan, Zeinab I. El Sayed, Mahmoud M. Abdel-Aal, Wael M. Abdel-Mageed, Eman Abdelsalam, Sahar Abdelaziz

**Affiliations:** 1 Department of Chemistry, College of Science, Princess Nourah Bint Abdulrahman University, P.O. Box 84428, Riyadh 11671, Saudi Arabia; msalsaleem@pnu.edu.sa; 2 Department of Pharmacognosy, Faculty of Pharmacy, Zagazig University, Zagazig 44519, Egypt; wafaahbh@zu.edu.eg (W.H.B.H.); zainb_ebrahim@zu.edu.eg (Z.I.E.S.); Mahmoud.ibrahim@su.edu.eg (M.M.A.-A.); eaamer@zu.edu.eg (E.A.); 3 Department of Pharmacognosy, College of Pharmacy, King Saud University, P.O. Box 2457, Riyadh 11451, Saudi Arabia; 4 Department of Pharmacognosy, Faculty of Pharmacy, Assiut University, Assiut 71526, Egypt

**Keywords:** UPLC-ESI-MS/MS, marine sponge-derived fungi, *Penicillium chrysogenum*, *Cliona* sp., antimicrobial, antioxidant, cytotoxicity

## Abstract

Marine sponge-derived endozoic fungi have been gaining increasing importance as promising sources of numerous and unique bioactive compounds. This study investigates the phytochemical profile and biological activities of the ethyl acetate extract of *Penicillium chrysogenum* derived from *Cliona* sp. sponge. Thirty-six compounds were tentatively identified from *P. chrysogenum* ethyl acetate extract along with the kojic acid (KA) isolation. The UPLC-ESI-MS/MS positive ionization mode was used to analyze and identify the extract constituents while 1D and 2D NMR spectroscopy were used for kojic acid (KA) structure confirmation. The antimicrobial, antioxidant, and cytotoxic activities were assessed in vitro. Both the extract and kojic acid showed potent antibacterial activity against *Staphylococcus aureus* and *Pseudomonas aeruginosa* with MIC 250 ± 0.82 µg/mL. Interestingly, the extract showed strong antifungal activity against *Candida albicans* and *Cryptococcus neoformans* with MIC 93.75 ± 0.55 and 19.53 ± 0.48 µg/mL, respectively. Furthermore, KA showed the same potency against *Fusarium oxysporum* and *Cryptococcus neoformans* with MIC 39.06 ± 0.85 and 39.06 ± 0.98 µg/mL, respectively. Ultimately, KA showed strong antioxidant activity with IC_50_ 33.7 ± 0.8 µg/mL. Moreover, the extract and KA showed strong cytotoxic activity against colon carcinoma (with IC_50_ 22.6 ± 0.8 and 23.4 ± 1.4 µg/mL, respectively) and human larynx carcinoma (with equal IC_50_ 30.8 ± 1.3 and ± 2.1 µg/mL, respectively), respectively. The current study represents the first insights into the phytochemical profile and biological properties of *P. chrysoenum* ethyl acetate extract, which could be a promising source of valuable secondary metabolites with potent biological potentials.

## 1. Introduction

The oceans are home to a tremendous diversity of species and their inhabitants produce a wealth of natural products [1] that are intertwined in intra-specific and inter-specific chemical communication [2]. Marine sponges, which are also known as microbial fermenters, are outstanding sources of highly diverse microbial communities, including new fungal species. The true producers of natural compounds from marine invertebrates are symbiotic microorganisms, which account for approximately 40 percent of the sponge’s volume [3,4,5]. The distinct pore structure of the sponges makes them an ideal host for a wide variety of marine microorganisms, which account for a large portion of sponge biomass [6]. Several sponge-derived fungi produce secondary metabolites which play vital biological roles as growth regulators, virulence factors, defense mechanisms, and communication signals with other organisms, in addition to their cytotoxic, antimicrobial, antioxidant, antiplasmodial, anti-hypercholesterolemic, and neuroprotective activities. Some of these compounds showed even stronger activities than the standard compounds [6,7].

The marine environment’s unique characteristics, such as hyper-salinity and high pressure, result in the formation of unique compounds by marine-derived fungi, with more variable structures than those formed by terrestrial species [8]. Recently, the presence of many fungal metabolites in the pharmaceutical market has indicated the potential of microorganisms as valuable sources of lead drugs. Notably, more than 253 studies in the literature reported around 774 new compounds, which were classified into nine classes, including alkaloids, peptides, terpenes, polyketides, macrolides, meroterpenoids, steroids, phenolics, and miscellaneous compounds [6]. Additionally, relevant reviews emphasized microbial metabolites as targets for the discovery and development of new drugs, mostly anticancer, antibiotic, antifungal, and antiparasitic drugs [9,10,11,12].

Interestingly, *Aspergillus* and *Penicillium*, which were obtained from a variety of sponge species, account for 25 percent of the total reported microbes displaying distinct chemical diversity [6]. In particular, *Penicillium chrysogenum* is a filamentous fungus that belongs to the genus *Penicillium*. In nature, it is a widely distributed mold, which is often found in food products and indoor environments. Moreover, it is the second most common genus of marine fungi [13]. It has previously been identified as the source of many ß-lactam antibiotics, most notably penicillin and other variable bioactive compounds with antimicrobial and anticancer activities [14].

Reports about the endophytic fungi from *Cliona* and *Hymedesmia sp* marine sponges have been lacking. Therefore, the aim of this study is to investigate, for the first time, the culturable fungal community associated with these Red Sea sponges, the characterization of the promising marine sponge-associated fungus *Penicillium chrysogenum*, UPLC-ESI-MS/MS analysis of its chemical constituents, and finally, the assessment of the antimicrobial, anticancer, and antioxidant potential of its metabolites.

## 2. Results and Discussion

### 2.1. Isolation and Screening of Endozoic Fungi with Potent Antimicrobial Activity from the Medicinal Marine Sponge

Two marine sponges, namely, *Cliona* sp. and *Hymedesmia* sp., were used as the source of endozoic fungi. In total, twelve fungal isolates were recovered from the marine sponges; four isolates were derived from *Cliona* sp. (*Aspergillus orchaceous*, *Aspergillus terreus*, *Aspergillus niger*, and *Penicillium Chrysogenum*), and eight isolates were recovered from *Hymedesmia* sp. (*Aspergillus terreus*, *Aspergillus awamori*, *Aspergillus niger*, *Aspergillus oryzae*, *Alternaria alternata*, *Trichoderma viridae*, *Penicillium lilacinum*, and *Aspergillus astus*) using malt yeast agar and malt extract agar media (Table 1). These fungal isolates were identified based on their species level according to the universal morphological keys [15,16,17]. The frequency of the genus *Aspergillus* is about 54 percent, whereas the frequency of the genus *Penicillium* is about 30 percent among the recovered endozoic fungi. The recovered isolates were assessed for their antimicrobial activities by inducing their growth on Wickerham’s broth medium, incubating them at standard conditions, and extracting with ethyl acetate. The agar dilution method was used to evaluate the antimicrobial activity based on the inhibition zone and minimum inhibitory concentration (MIC) determination. De Oliveira et al. [18] and Sartoratto et al. [19] classified the antimicrobial activity of the extract based on the MIC values as follows: 50–500 µg/mL = strong activity; 600–1500 µg/mL = moderate activity; and >1500 µg/mL = weak activity or inactivity. Consequently, from the screening profile as illustrated in Table 1, the highest antimicrobial activity was reported for the *Penicillium chrysogenum* (C6) extract that showed a strong antibacterial activity against *Staphylococcus aureus*, with MIC value equivalent to 250 ± 0.82 µg/mL, and superior fungicidal activity against *Candida albicans*, with MIC 93.75 ± 0.78 µg/mL. Furthermore, *Aspergillus terreus* (H2) from *Hymedesmia* sp. is the second fungal isolate, which revealed potent antibacterial activity against *Staphylococcus aureus* and strong antifungal activity against *Candida albicans*, with MIC 250 ± 1.5 µg/mL and 187.5 ± 0.95 µg/mL, respectively. Interestingly, *Aspergillus oryzae* (H5) from *Hymedesmia* sp. exhibited strong antibacterial activity against highly resistant Gram-negative bacteria *Pseudomonas aeruginosa* and moderate fungicidal activity against *Candida albicans*, with MIC values equivalent to 250 ± 1.3 µg/mL and 750 ± 0.88 µg/mL, respectively. The remaining fungal isolates from both tested marine sponges (Table 1) displayed weak to negative antimicrobial activities against the tested organisms.

### 2.2. Characterization of Penicillium Chrysogenum Ethyl Acetate Extract (PC) and the Isolated Kojic Acid (KA)

Recently, marine-derived endophytic fungi investigation has drawn the attention of many researchers; as such, fungi are considered to comprise an unexplored repertoire of unprecedented bioactive compounds. The rationale behind the future use of endophytic fungi to produce biologically active secondary metabolites arises from their rapid growth on culture medium, bulk biomass production accessibility, and independence from environmental variables. *Penicillium* is one of the most isolated fungi from the marine environment. It has been proven that the *Penicillium* species comprise valuable biological resources, as they produce important enzymes and secondary metabolites. *Penicillium* is a decomposer of various marine organisms and play an essential role in pollutant degradation and nutrient recycling [20]. The recovered *P. chrysogenum* fungal isolate from *Cliona* sp. (GenBank accession no. OL597937.1) was screened for its phytochemical constituents, using UPLC-ESI-MS/MS analysis, and its biological activities.

#### 2.2.1. Spectroscopic Analyses of the Isolated Kojic Acid

The UPLC-ESI-MS/MS (positive mode) of KA (R_t_. 1.13 min, 4) showed a protonated molecular ion peak at *m/z* 143 [M + H]^+^, thereby confirming the compound molecular formula C_6_H_6_O_4_, in addition to fragment ion peaks at 113 amu [M + H-CH_2_OH]^+^, 97 [113 –OH]^+^, and 69 [97 –CO]^+^, as shown in Figure S1 [21]. The IR υ_max_ (KBr) spectrum showed absorption bands at 3142, 2923, and 1656 cm^−1^, thereby indicating the presence of hydroxyl, alkene, and carbonyl groups, respectively (Figure S2).

The ^1^HNMR (400 MHz, DMSO-*d_6_*) spectrum showed two protons at δ_H_ 6.33 (1H, s, H-3) and 8.02 (1H, s, H-6) ppm, corresponding to vinylic protons [22] (Figure S3 and Table S1). The ^13^CNMR (100 MHz, DMSO-*d_6_*) spectrum (Figure S4) showed three quaternary carbons at δ_c_ 173.9, 168.1, and 139.3, which corresponded to C-4, C-2, and C-5, respectively, in addition to two olefinic carbons (C-6 and C-3) at δ_c_ 145.7 and 109.8, respectively, and one methylene carbon (C-7) (*δ*c 59.5). The ^1^H and ^13^C NMR data in combination with the ^1^H-^13^C HSQC and ^1^H-^13^C HMBC analyses confirmed the presence of a pyrone ring [22] (Figures S5–S7). All these data are in good agreement with those previously reported [22,23].

#### 2.2.2. Structural Identification of Constituents of *P. chrysogenum* Ethyl Acetate Extract by UPLC-ESI-MS/MS

**Figure 1:**
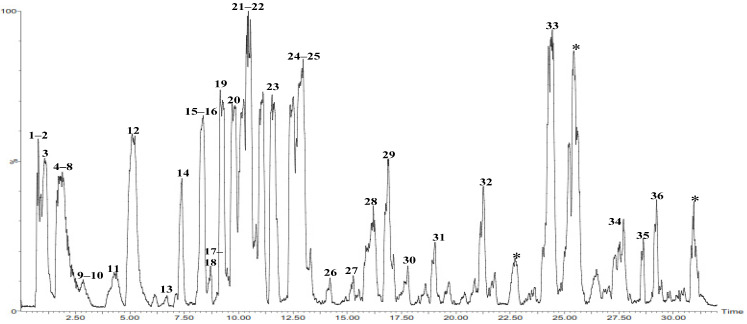
UPLS-ESI-MS chromatogram of *Penicillium chrysogenum* ethyl acetate extract in positive (+) ionization mode. * Unidentified compounds.

In the current study, UPLC-ESI-MS/MS in the positive ion mode was used to analyze the ethyl acetate extract of *P. chrysogenum* (PC) for the first time. The compounds’ identification was based on their MS^2^ data, which was provided by the precursor ion mass and their fragments, neutral mass loss, and comparison with the available literature. The compounds were arranged based on their retention time (R_t_), as shown in Table 2 and Figure 1. When using the positive mode in LC-MS analyses, the pseudo molecular ion [M + H]^+^ was not the common MS signal, water losses were very common ([M + H-H_2_O]^+^), and alkali adducts (mainly [M + Na]^+^) also regularly existed. In total, 36 compounds were tentatively identified in the *P. chrysogenum* extract. Table 2 and Figure 1 indicate the tentatively identified compounds along with their retention times, experimental *m/z* in positive ionization mode, and MS/MS fragments.

**Figure 2:**
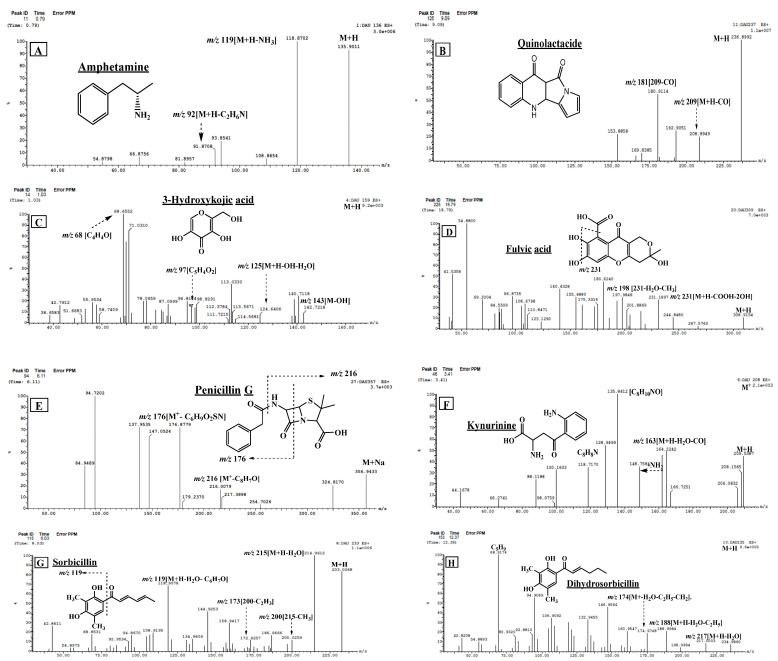

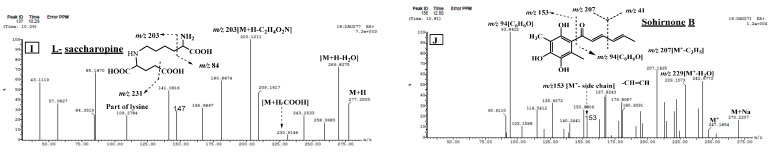
UPLC-ESI-MS/MS chromatograms of some identified compounds ((**A**): amphetamine; (**B**): quinolactacide; (**C**): 3- hydroxy kojic acid; (**D**): fulvic acid; (**E**): penicillin G; (**F**): Kynurenine; (**G**): sorbicillin; (**H**): dihydrosorbicillin; (**I**): L- saccharopine; (**J**): sohirnone B) in *Penicillium chrysogenum* ethyl acetate extract in positive (+) ionization mode.

Compound **1** (R_t_. 0.79 min) was tentatively assigned as amphetamine. The ESI-MS spectrum showed a protonated molecular ion peak at *m/z* 136.0710 [M + H]^+^ in addition to a daughter ion at *m/z* 119 [M + H-NH_3_]^+^ with loss of NH_3_ moiety, and a fragment ion at *m/z* 92, which showed the loss of C_2_H_6_N leaving the benzyl moiety. The loss of the fragment C_2_H_6_N was confirmed by the presence of the fragment ion at *m/z* 54 (Figure 2A). From this fragmentation pattern and through comparison with the literature, compound **1** was concluded to be amphetamine [24]. Amphetamine is a psychostimulant drug of phenyl alanine class that is present in two enantiomers, d- and l-enantiomers. The d-enantiomer is more active than the l-form and is used for treating narcolepsy and as diet pills. It is noteworthy to say that amphetamine was used to promote wakefulness in the soldiers during the Second World War [25].

Compounds **2** and **7** (R_t_. 0.88 and 1.84 min) showed di-protonated molecular ion peaks [M + 2H]^+^ at *m/z* 115.0515 and 115.0537, respectively, with a daughter ion at *m/z* 113 [M^+^]. MS^2^ fragmentation also showed fragment ions at *m/z* 96 [M + H-H_2_O]^+^ and 69 [M + H-COOH]^+^ with the losses of one molecule of water and carboxylic acid moiety, respectively. The fragment at *m/z* 41 showed loss of CH_2_N from the fragment ion (*m/z* 69), leaving C_3_H_5_ moiety. From ESI/MS/MS fragmentation and by comparison with the literature, compounds **2** and **7** were tentatively identified as pyrroline carboxylic acid and its isomer [26]. Notably, it was previously identified from *P. citrinum* [27].

Compound **3** (R_t_. 1.03 min) was tentatively identified as 3-hydroxy KA (Figure 2C). The ESI-MS spectrum showed a molecular ion peak at *m/z* 159.1239 [M + H]^+^ and an MS^2^ fragment ion at *m/z* 143, assigned for (KA + H^+^). The remaining fragment ions, at *m/z* 125 [M + H-OH-H_2_O]^+^, 113 [M^+^ + H-OH-CH_2_O]^+^, 96 [M^+^ + H-OH-CH_2_O-H_2_O]^+^, and *m/z* 68 [M + H-H_2_O-OH-CO-CH_2_O]^+^, showed similar fragmentation patterns as KA, with the exception of the presence of the additional hydroxyl group [28].

Compound **4** (R_t_. 1.13 min) showed a protonated molecular ion peak [M + H]^+^ at *m/z* 143.0706, and was tentatively identified as kojic acid [21,22]. Kojic acid, a pyrone derivative, is a major secondary metabolite produced by a limited range of microorganisms, including *Aspergillus oryzae*, *Aspergillus flavus*, and *Aspergillus tamarii*, as well as *Penicillium* species and certain bacteria in the stationary phase of growth. The most important biological functions of KA are antibacterial, fungicidal, and insecticidal activities, and it has been approved as a food and cosmetic additive. Furthermore, KA can act as a tyrosinase inhibitor (inhibition of melanin formation) by chelating copper atoms at the tyrosinase active site. For clinical trials, the 1 percent KA-containing formulation was shown to be effective for treating hyperpigmentation disorders, such as melisma, post-inflammatory hyperpigmentation, age spots, and freckles. KA is now accepted to be safe as a cosmetic ingredient and continues to be used as a skin lightening product [29,30]. Notably, KA was previously isolated from *P.* *chrysogenum* endophyte of *Strychnos toxifera*, which is a poisonous plant [31].

Compound **5** (R_t_. 1.20 min) analysis generated ESI-MS at *m/z* 185.1388 [M + H]^+^ and MS^2^ fragmentation at *m/z* 167, which was produced by the loss of a water molecule. The daughter ion at *m/z* 139 showed subsequent loss of the carbonyl group. Furthermore, the fragment ions at *m/z* 125, 95, and 80, which showed the loss of CH_2_, CH_2_O, and CH_3_, respectively, were also observed in the MS^2^ spectrum. Thus, compound **5** was identified as aspyrone according to the previously reported data [21]. It was reported that aspyrone showed nematocidal activity toward *Pratylenchus penetrans* [32].

Compound **6** (R_t_. 1.35 min) generated ESI-MS at *m/z* 143.0601 [M + H]^+^ and MS^2^ fragmentation at *m/z* 125, which was produced by the loss of a water molecule. On the other hand, the daughter ion at *m/z* 113 showed subsequent loss of the CH_2_OH group. Furthermore, the fragment ion at *m/z* 96 [113-OH]^+^ was observed in the MS^2^ spectrum with the base peak fragment at *m/z* 69 [97-CO]. Thus, compound **6** was tentatively identified as fulfuran [7].

Compound **8** (R_t_. 2.15 min) ESI-MS^1^ showed a molecular ion peak at *m/z* 208.0558 [M + H]^+^. The MS^2^ fragmentation of **8** showed loss of the methyl amine group leaving the fragment ion at *m/z* 177. Furthermore, the loss of C_4_H_8_O was evidenced from the fragment ion at *m/z* 136 in addition to the fragment ion at *m/z* 85, which corresponded to C_5_H_11_N. Compound **8** was tentatively identified as *N*-methyl benzodioxazoyl butan amine (MBDB) [24] from this fragmentation pattern and through comparison with the literature.

Compound **9** (R_t_. 3.35 min) ESI-MS showed a molecular ion peak at *m/z* 185.1051 [M + H]^+^. The MS^2^ fragmentation of this compound showed the loss of the hydroxyl group leaving the fragment ion at *m/z* 158. The loss of C_2_H_4_O was evidenced from the fragment ion at *m/z* 141, which, in turn, resulted in the loss of the carbonyl group, thereby facilitating the major fragment ion at *m/z* 113 [141-CO]^+^. Furthermore, the fragment ion at *m/z* 98 showed another subsequent loss of CH_3_. Moreover, the fragment at *m/z* 128 showed the loss of C_3_H_5_O moiety of the tricyclic ring. Compound **9** was tentatively identified as asperlactone from this fragmentation pattern and through comparison with the literature [21].

Compound **10** (R_t_. 3.53 min) analysis showed a molecular ion peak at *m/z* 185.0992 [M + H]^+^ and a fragment ion at 143 (KA + H^+^), thereby showing the loss of the acetyl group. Furthermore, the remaining fragment ions at *m/z* 125 [M + H-H_2_O-COCH_3_]^+^, 113 [M + H-COCH_3_-CH_2_O]^+^, 97 [M + H-COCH_3_-CH_2_O-H_2_O]^+^, and 68 [M + H-COCH_3_-CO-CH_2_O-H_2_O]^+^ showed the same fragmentation pattern as that of KA, thereby suggesting the tentative identification of this compound as acetyl KA [21].

Compound **11** (R_t_. 4.83 min) generated a molecular ion peak [M + H]^+^ in ESI-MS at *m/z* 141.0650. It also gave the diagnostic fragment ion at *m/z* 123 [M + H-H_2_O]^+^, which corresponded to the loss of a water molecule followed by the loss of an ion fragment at *m/z* 95 [123-CO]^+^ and 67 [95-CO]^+^. This showed losses of two molecules of the carbonyl group from the protonated ion. Compound **11** was identified as KA aldehyde by comparison with literature [33].

Compounds **12** and **14** (R_t_. 6.11 and 7.87 min) were tentatively identified as penicillin G and its stereoisomer from ESI-MS^1^ and MS^2^ spectral data (Table 2). The MS^2^ spectrum (Figure 2E) showed a prominent molecular ion peak at *m/z* 356.1262 and 356.9433 [M + Na]^+^, respectively, and fragment ions at *m/z* 217 [M + H-C_8_H_7_O]^+^ and *m/z* 176 [M + H-C_6_H_9_O_2_NS]^+^, which showed the loss of the thiazolidine ring. The mass data of these compounds are in good agreement with those in the literature of penicillin G along with its stereoisomers [34]. Penicillin G was also isolated from *P. chrysogenum* endophytic fungus on the mangrove plant *Porteresia coarctata* (Roxb.) [35].

Compound **13** (R_t_. 6.48 min) gave a molecular ion peak at *m/z* 241.1167 [M + H]^+^. The ESI-MS/MS fragmentation pattern showed fragment ions at *m/z* 212 [M + H-NHCH_2_]^+^ and 171 [M + H-C_3_H_5_NO]^+^, in addition to the fragment at *m/z* 69. This corresponded to the imidazole ring C_4_H_4_N_2_, which is in good agreement with that of anserine reported by Peiretti et al. [36].Consequently, this compound was identified to be anserine [26,36].

Compound **15** (R_t_. 8.02 min) generated a protonated molecular ion peak at *m/z* 193.1551 [M + H]^+^. It also facilitated a diagnostic MS^2^ fragment ion at *m/z* 139 [M+ H-C_2_H_3_NO]^+^ that corresponded to the neutral losses of acetyl and NH moieties. A prominent base peak fragment ion at *m/z* 104 [M + H-C_4_H_8_S]^+^ was also detected. Moreover, other fragment ions, [M+H-H_2_O-CH_3_]^+^ and [M + H-H_2_O-CH_3_-CO]^+^ at *m/z* 160 and *m/z* 115, respectively, were also detected. According to the literature, compound **15** was tentatively identified as *N*- acetyl methionine [37].

Compound **16** (R_t_. 8.03 min) was tentatively identified as sorbicillin from ESI-MS (Figure 2G), which showed a protonated molecule ion peak at *m/z* 233.1105 [M + H]^+^ with an MS^2^ base peak fragment ion at *m/z* 215 [M-H_2_O]^+^. In addition, the fragment ions at *m/z* 200 and 173 exhibited subsequent losses of the CH_3_ and C_2_H_3_ groups, respectively. Notably, a prominent fragment peak was observed at *m/z* 119 that corresponded to [M + H-H_2_O]^+^ with the loss of the side chain [C_6_H_7_O]^+^. From this fragmentation pattern and through comparison with literature, the compound was concluded to be sorbicillin, which was previously isolated in addition to dihydrosorbicillin from *Penicillin chrysogenum* [13]. Sorbicillin was also isolated from *Penicillium* sp. endophyte of Chinese mangrove *Bruguiera gymnorrhiza* [38].

Compound **17** (R_t_ 8.55 min) showed a molecular ion peak at *m/z* 335.1497 [M + H]^+^ and a daughter ion at *m/z* 155 [M + 3H-C_9_H_10_O_4_]^+^, which corresponded to the loss of [tropylium cation + 2COOH]^+^. According to the available literature, compound 17 was tentatively assigned as penillic acid. To the best of our knowledge, this compound was determined to be a degradation product of penicillin G [39].

Compounds **18** and **20** (R_t_. 8.82 and 10.29 min) exhibited molecular ion peaks in the ESI/MS spectrum at *m/z* 277.0930 and 277.2620 [M + H]^+^, respectively. The MS^2^ fragment ion at *m/z* 259 showed the neutral loss of a water molecule. In addition, the base peak fragment at *m/z* 203 indicated the loss of C_2_H_4_O_2_N, whereas the fragment at *m/z* 231 showed the neutral loss of the COOH group. The presence of the lysine amino acid in this compound was clear from the presence of two fragment ions at *m/z* 147 and 84 (Figure 2I). According to this fragmentation and through comparison with literature, these compounds were tentatively identified as L-saccharopine and its isomer [26].

Compound **19** (R_t_. 9.09 min) was identified as quinolactacide from ESI-MS^1^ and MS^2^ spectral data (Figure 2B), as shown in Table 2. It facilitated a protonated molecular ion peak at *m/z* 237.1015 [M + H]^+^ [100 percent relative abundance] and diagnostic fragment ions at *m/z* 209 [M + H-CO]^+^ and 181 [209-CO]^+^, which showed the loss of two carbonyl groups. By comparison with the literature, it was confirmed that this compound could be named quinolactacide. Notably, quinolactacide has an excellent insecticidal activity against peach aphids. It is noteworthy to say that quinolactacide was previously isolated from *Penicillium citrinum* [40].

Compounds **21** and **22** (R_t_. 10.38 and 10.98 min) showed the same protonated molecular ion at *m/z* 349.1757 and 349.2006 [M + H]^+^, respectively, in addition to an MS^2^ fragment ion at *m/z* 305 [M + H-44]^+^, which resulted from the loss of 44 amu that corresponded to the neutral loss of CO_2_. Other fragment ions were observed at 277 [M + H-CO_2_-C_2_H_4_]^+^, 249 [M + H-CO_2_-C_2_H_4_-CO]^+^, and 221. The first two fragments were generated from the subsequent neutral loss of C_2_H_4_ and CO, respectively, whereas the third fragment *m/z* 221 may have been generated from the molecular skeleton fragmentation and rearrangement. Compounds **21** and **22** were tentatively assigned as camptothecin and its isomer from the previous ESI-MS/MS fragmentation and based on the available literature [41,42].

Compound **23** (R_t_. 12.37 min) generated its ESI-MS at *m/z* 235.2265 [M + H]^+^ and MS^2^ fragment ions at *m/z* 217 [M + H-H_2_O]^+^, 188 [M + H-H_2_O-C_2_H_5_]^+^, and 174 [M + H-H_2_O-C_2_H_5_-CH_2_]^+^, in addition to a base peak fragment ion at *m/z* 69, which showed loss of C_5_H_9_ moiety (Figure 2H). These fragment ions, in addition to other fragment ions at *m/z* 160, 147, 133, 106, and 94, indicated that this compound is related to sorbicillin and, in turn, this compound was concluded to be dihydrosorbicillin [13]. Notably, dihydrosorbicillin was previously identified by *P. chrysogenum* [43].

Compound **24** (R_t_. 12.81 min) was tentatively identified as sohirnone B. It belongs to the class of organic compounds known as sorbicillinoides. It gave molecular ion peaks [M + Na]^+^ at *m/z* 271.1547 and 247 [M]^+^ (Figure 2J). MS^2^ showed a fragment ion at *m/z* 229 [M-H_2_O]^+^, which was produced by the loss of one molecule of water. In addition, the daughter ion at *m/z* 207 [M-C_3_H_5_]^+^ resulted from the loss of CH_3_-CH=CH moiety. The next fragment ion at *m/z* 181 showed loss of CH=CH moiety. The fragment ion at *m/z* 153 detected the loss of side chain moiety [CH_3_-CH=CH-CH=CH-C=O]^+^. This fact was confirmed from the base peak fragment at *m/z* 94, which corresponded to the side chain. Therefore, compound **24** was identified as sohirnone B, as previously published [38]. Sohirnone B was reported to have weak antibacterial activity on *Staphylococcus aureus* and *Bacillus subtilis* [44]. Sohirnone B was previously identified from *P. chrysogenum* [45].

Compound **25** (R_t_. 12.97 min) was tentatively identified as kynurenine along with its isomers, as the ESI/MS^1^ shows a protonated molecular ion peak at *m/z* 209.2372 [M + H]^+^. The ESI-MS^2^ fragment ions at *m/z* 164 and 148 showed the loss of the COOH and NH_2_ groups, respectively. The base peak fragment ion at *m/z* 136 showed the loss of C_2_H_3_NO_2,_ and this loss was followed by the loss of one molecule of water to facilitate a fragment ion at *m/z* 118. This fragmentation pattern along with the other fragments shown in Figure 2F were in a good agreement with that of kynurenine [26,46]. To the best of our knowledge, kynurenine is found in the human lens, and it is thought to play an important role in protecting the lens and retina from UV-induced photodamage, reducing chromatic aberration, and sharpening the image on the retina [46].

Compound **26** (R_t_ 14.42 min) ESI-MS showed a molecular ion peak [M + H]^+^ at *m/z* 309.1964. Furthermore, the daughter ion was observed at *m/z* 217, and was produced after the loss of benzyl moiety, followed by the loss of the carbonyl group to facilitate the fragment ion at *m/z* 189. Subsequently, this resulted in the loss of cyanide moiety to facilitate a major fragment ion at *m/z* 159. Moreover, the loss of C_6_H_8_O_2_NS facilitated a fragment ion at *m/z* 148. From this ESI-MS/MS fragmentation and according to the available literature, the compound **26** was tentatively assigned as penilloic acid [47]. It is noteworthy to say that this compound could be produced by the exposure of penicillin G to an alkaline medium [39].

Compound **27** (R_t_. 15.17 min) gave a molecular ion peak [M + H]^+^ at *m/z* 475.2501 and MS^2^ fragment ions at *m/z* 248 [sohirnone B + H]^+^. Furthermore, daughter ions at *m/z* 207 and 180 (as previously mentioned in sohirnone B) were also detected. Thus, compound **27** was tentatively identified to be a sohirnone B derivative [38].

Compounds **28** and **29** (R_t_ 16.08 and 17.02 min) were identified as sorrentenone and its isomer from ESI-MS^1^ and MS^2^ spectral data (Table 2), respectively. Molecular ion peaks [M + Na]^+^ at *m/z* 269.1486 and 269.1831, respectively, were also detected. The MS^2^ spectra of the two molecules showed a fragment ion at *m/z* 250 [M + H-H_2_O]^+^, which corresponded to the loss of an H_2_O molecule. In addition, fragment ions at *m/z* 155 [250-C_5_H_7_O]^+^ explain the loss of the side chain C_4_H_7_ and the carbonyl group. The mass data of these compounds are in good agreement with that of sorrentenone reported in the literature [44].

Compound **30** (R_t_. 18.79 min) showed a protonated molecular ion [M + H]^+^ at *m/z* 309.1964 followed by a daughter ion peak at *m/z* 231 [M + H-CO_2_-2OH]^+^, which corresponded to the loss of the carboxyl group and two hydroxyl groups. Furthermore, fragment ions at *m/z* 198 and 181 resulted from the cleavage of the ring B (Figure 2D). The mass information about this compound is in good agreement with the previously published data [21]. Fulvic acid is a phenolic acid and a fungal metabolite that was originally isolated from the *Penicillium* species. It acts as an immune modulator, reduces oxidative stress, induces apoptosis in hepatic cancer cell lines, influences the microbiome, and potentially affects gut health. Also, fulvic acid decreases proinflammatory markers and activates the immune system to kill bacteria [48]. Fulvic acid was previously identified from *Penicillium* sp. [49].

Compound **31** (R_t_ 19.72 min) ESI-MS showed a prominent sodiated molecular ion peak [M + Na]^+^ at *m/z* 273.2188, in addition to a sodium-cationized molecule at *m/z* 255 [M + Na-H_2_O]^+^, thereby showing the loss of one molecule of water. Several other fragments were produced, including *m/z* 227 [M + Na-HCOOH]^+^, 119, 115, and 91. Compound **31** was tentatively assigned as citrinin from this fragmentation and by comparison with the available literature [50,51]. Citrinin is a polyketide mycotoxin with the molecular formula C_13_H_14_O_5_. It was first isolated as a pure compound from a culture of *Penicillium citrinum* [52]. It is mainly produced by *Aspergillus, Penicillium,* and *Monascus* fungi. Citrinin is a mycotoxin that contaminates various commodities of plant origin and it is nephrotoxic, genotoxic, and carcinogenic in nature [51,53]. Citrinin was previously isolated from *P. chrysogenum* [54].

Compound **32** (R_t_ 21.98 min) was identified as zearalenone from MS^1^ and MS^2^ spectral data (Table 2). The MS^2^ spectrum of **32** showed a molecular ion peak [M + H]^+^ at *m/z* 319.2695 and fragment ions at *m/z* 283 [M + H-2H_2_O]^+^, corresponding to the loss of two H_2_O molecules, and daughter ions were also detected at *m/z* 98, 83, 59 and 42 [55]. It was also produced by *Penicillium* sp. [56].

Compound **33** (R_t_. 24.21 min) gave a molecular ion peak at *m/z* 377.2721 [M + H-H_2_O]^+^, thereby showing the loss of one water molecule. An MS^2^ fragment at *m/z* 252 [M + H-H_2_O-C_9_H_17_]^+^ was observed and showed the loss of the side chain. Furthermore, other fragments at *m/z* at 189, 156, and 134 were detected. This fragmentation helps in tentative identification of compound **33** as dehydroergosterol, as previously published [57]. Dehydroergosterol might be helpful in slowing down the deterioration of brain function by inducing a suitable microglial phenotype. However, the permeability of DHE through the blood–brain barrier was not assessed. DHE is considered safe to consume, and it is present in various dairy products, such as camembert cheese. Thus, DHE might be a valuable preventive tool for dementia [58]. It was also isolated from *Penicillium roqueforti* [59].

Compound **34** (R_t_. 27.47 min) exhibited a molecular ion peak at *m/z* 395.3597 [M + H-H_2_O]^+^, with daughter ions at *m/z* 255, which show the loss of a side chain and 214 [M + H-H_2_O-C_13_H_25_]^+^, corresponding to the loss of side chain and ring D cleavage. Compound **34** was tentatively identified as stigmasterol from these data and with the aid of the present literature [60,61]. Stigmasterol is an efficient antifungal and broad-spectrum antibacterial agent. As such, it may be used as a valuable compound in the development of novel antimicrobial drugs [62]. Stigmasterol was previously identified from *Penicillium crusosum* Thom [63].

Compound **35** (Rt. 27.70 min) was identified as Ergosta-4,6,8(14),22-tetraen-3-one from MS^1^ and MS^2^ spectral data. A protonated molecular ion peak at *m/z* 393.3486 [M + H]^+^ was formed, and a daughter ion peak at *m/z* 335 [M + H-CO-2CH_3_]^+^ was observed, corresponding to the loss of the carbonyl group and two methyl groups in addition to the fragment ion at *m/z* 268 [M + H–C_9_H_17_]^+^, which corresponded to the loss of the side chain. Moreover, remaining fragments at m/z 173, 69, and 55 were also detected. Hence, compound **35** was identified as Ergosta-4,6,8(14),22-tetraen-3-one by comparison with the available literature [64]. Ergosta-4,6,8(14),22-tetraen-3-one was previously isolated from the terrestrial *Penicillium ssp*. [65].

Compound **36** (Rt. 29.91) generated its ESI-MS at *m/z* 639.6279 [M + H]^+^. Moreover, MS^2^ exhibited daughter ions at *m/z* 381 [M + H-C_16_H_32_O_2_]^+^, thereby showing the loss of palmitic acid facilitating a molecular ion of brassicasterol. Compound **36** was tentatively identified as brassicasterol palmitate by comparison with the literature [60].

Interestingly, this is the first report on the presence of amphetamine (1), 3-hydroxy kojic acid (3), aspyrone (5), fulfuran (6), asperlactone (9), acetyl kojic acid (10), kojic acid aldehyde (11), anserine (13), *N*-acetyl methionine (15), L-saccharopine (18), quinolactacide (19), camptothecine (21), kynurenine (25), penilloic acid (26), and brassicasterol palmitate (36) from *penicillium* sp., while other compounds were previously reported either from *P. chrysogenum* or from other *Penicillium* sp.

**Table 2:** Phytochemicals tentatively identified in *Penicillium chrysogenum* ethyl acetate extract using UPLC-ESI-MS/MS analysis in positive ionization mode.

No.	Compound Name	R_t_ (min.)	Parent ion *(m/z)*	MS^2^ Fragments *(m/z)*	Area% Total	Reference
1	Amphetamine *	0.79	136.0710 [M + H]^+^	119, 92, 54	1.51	[24,25]
2	Pyrroline carboxylic acid	0.88	115.0515 [M + 2H]^+^	96, 69, 41	0.50	[26]
3	3-hydroxy kojic acid *	1.03	159.1239 [M + H]^+^	143, 125, 113, 96, 69	0.18	[28]
4	Kojic acid	1.13	143.0706 [M + H]^+^	126, 113, 97, 69	2.87	[21,22]
5	Aspyrone *	1.20	185.1388 [M + H]^+^	167, 139, 125	0.50	[21]
6	Flufuran *	1.35	143.0601 [M + H]^+^	125, 113, 96, 69	0.59	[7]
7	Pyrroline carboxylic acid isomer	1.84	115.0537 [M + 2H]^+^	96, 69, 41	0.50	[26]
8	N-methyl-benzodioxazoylbutanamine (MBDB)	2.15	208.0558 [M + H]^+^	177, 136, 85	0.59	[24]
9	Asperlactone *	3.35	185.1051 [M + H]^+^	158, 141, 128, 113, 98	0.77	[21]
10	Acetyl Kojic acid *	3.53	185.0992 [M + H]^+^	143, 125, 113, 97, 68	0.50	[21]
11	Kojic acid aldehyde *	4.83	141.0650 [M + H]^+^	123, 95, 67, 43	5.92	[33]
12	Penicillin G	6.11	356.1262 [M + Na]^+^	217, 176	0.46	[34]
13	Anserine *	6.48	241.1167 [M + H]^+^	212, 171, 69	1.75	[26,36]
14	Penicillin G isomer	7.87	356.9433 [M + Na]^+^	217, 176	3.34	[34]
15	N-acetyl methionine	8.02	193.1551 [M + H]^+^	160, 139, 115, 104	4.05	[37]
16	Sorbicillin	8.03	233.1105 [M + H]^+^	215, 200, 173, 145, 119	0.24	[13]
17	Penillic acid	8.55	335.1497 [M + H]^+^	155	0.24	[39]
18	L-saccharopine *	8.82	277.0930 [M + H]^+^	259, 231, 215, 203, 147, 84	4.05	[26]
19	Quinolactacide *	9.09	237.1015 [M + H]^+^	209, 192, 181, 169, 154	0.24	[40]
20	L-saccharopine isomer	10.29	277.2620 [M + H]^+^	259, 231, 215, 203, 147, 84	0.15	[26]
21	Camptothecin *	10.38	349.1757 [M + H]^+^	305, 277, 249, 221	3.77	[41,42]
22	Camptothecin isomer	10.98	349.2006 [M + H]^+^	305, 277, 249, 221	3.38	[41,42]
23	Dihydrosorbicillin	12.37	235.2265 [M + H]^+^	217, 199, 188, 174, 160, 147, 133, 106, 94, 69 (100%).	4.51	[13]
24	Sohirnone B	12.81	271.1547 [M + Na]^+^	247, 229, 207, 181, 153, 94	0.31	[38]
25	Kynurenine *	12.97	209.2372 [M + H]^+^	164, 148, 136, 118, 94	7.95	[26,46]
26	Penilloic acid *	14.42	309.1964 [M + H]^+^	217, 189, 159, 148.	0.36	[47]
27	Sohirnone B derivative	15.17	475.2501 [M + H]^+^	248, 207, 180	0.27	[38]
28	Sorrentanone	16.08	269.1486 [M + Na]^+^	250, 208, 180, 155	3.72	[44]
29	Sorrentanone isomer	17.02	269.1831 [M + Na]^+^	250, 208, 180, 155	3.11	[44]
30	Fulvic acid	18.79	309.1964 [M + H]^+^	231, 198, 181	2.82	[21]
31	Citrinin	19.72	273.2188 [M + Na]^+^	255, 227, 119, 115, 91	0.61	[50,51]
32	Zearalenone	21.98	319.2695 [M + H]^+^	283, 98, 83, 59	0.55	[55]
33	Dehydro ergosterol	24.21	377.2721 [M + H-H_2_O]^+^	267, 252, 189, 156, 134	0.44	[57]
34	Stigmasterol	27.47	395.3597 [M + H-H_2_O]^+^	311, 255, 215	1.09	[60,61]
35	Ergosta-4,6,8(14),22 tetraen-3one	27.70	393.3486 [M + H]^+^	335, 268, 250, 173	1.11	[64]
36	Brassicasterol palmitate *	29.91	639.6279 [M + H-H_2_O]^+^	381	1.62	[60]

* Compounds first reported in *Penicillium chrysogenum_._*

### 2.3. Biological Activities of Penicillium Chrysogenum Ethyl Acetate Extract and Kojic Acid (KA)

#### 2.3.1. Antimicrobial Activity and MIC 

The discovery of novel classes of antimicrobial therapies has increased global concern over the span of the last few years. Many harmful microbes are now becoming resistant to many available antibacterial and antifungal drugs [66,67]. In the last few decades, interesting bioactive secondary metabolites from marine endophytic fungi have been reported with relevant clinical aims, some of which are matchless. The Red Sea, Egypt represents a diverse source of marine sponge-derived endophytic fungi. These organisms could be a potential host to a unique natural product [68]. The antibacterial and antifungal activity against different microorganisms by the well diffusion technique were expressed as the diameter of the inhibition zone and the percentage of activity, as shown in Table 3 and Table 4.

**Figure 3:**
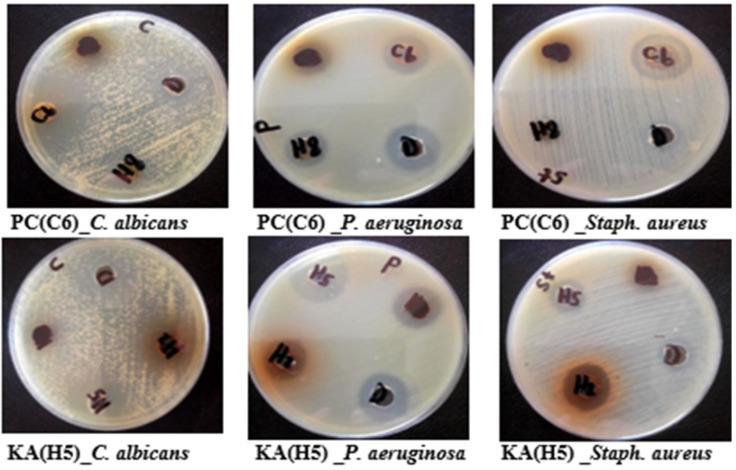
Antimicrobial activity of *P. chrysogenum* ethyl acetate extract PC(C6) and Kojic acid KA(H5) by agar dilution method with *S. aureus, P. aeruginosa,* and *C. albicans*.

The *P. chrysogenum* ethyl acetate extract showed strong antibacterial activity against *S. aureus* only with MIC value 250 µg/mL, with superior fungicidal activity against *Candida albicans* and *Cryptococcus neoformans* with MIC values equivalent to 93.75 and 19.53 µg/mL, respectively (Figure 3). Furthermore, the extract showed weak activity against *Aspergillus fumigatus* and *Aspergillus flavus*, with no activity against *Fusarium oxysporum*. Interestingly, KA also showed strong antibacterial activity against highly resistant gram-negative bacteria *Pseudomonas aeruginosa* with an MIC value equal to 250 µg/mL, and moderate fungicidal activity against *Candida albicans* with an MIC value equal to 750 µg/mL (Figure 3). The antimicrobial activity of the extract, based on the MIC values, was classified as follows: 50–500 µg/mL = strong activity; 600–1500 µg/mL = moderate activity; and >1500 µg/mL = weak activity or inactive [18].

#### 2.3.2. Antioxidant Activity

**Figure 4:**
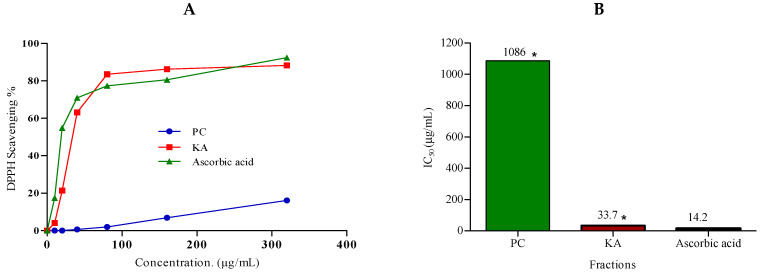
(**A**) The 2,2-diphyenyl-picrylhydrazyl (DPPH) radical scavenging activity of different concentrations (10–320 µg/mL) of *Penicillium chrysogenum* total extract (PC) and Kojic acid (KA). (**B**) IC_50_ of antioxidant activity of *Penicillium chrysogenum* total extract (PC), Kojic acid (KA), and ascorbic acid (positive control). DPPH in methanol (without the tested sample) was used as a negative control. Data were analyzed by using one-way ANOVA and statistical significance was calculated with Dunnett’s multiple comparisons test. * *p*-value < 0.05 was considered as statistically significant. The data display the mean ±SD of three biological replicas. Asterisk indicates a significant difference compared to the control (*p* < 0.01).

The series of concentrations ranged from 10 to 320 µg/mL in methanol of DPPH, and *P. chrysogenum* (PC) ethyl acetate extract, KA, and ascorbic acid were used in the current study. The DPPH scavenging percentage of PC extract and KA, as well as ascorbic acid and IC_50_ values, are presented in Figure 4A,B, respectively. All of them showed a concentration-dependent antioxidant activity, as demonstrated by the increase in their DPPH radical scavenging activity.

The tested fraction and/or compound is considered as a very strong antioxidant when IC_50_ values are <50 and is considered strong (50–100), moderate (100–150), or weak (151–200) µg/mL [69]. According to the previous range of IC_50_ values, the total extract exhibited very weak antioxidant activity with IC_50_ 1086.2 ± 34.6, whereas pure KA showed a strong activity, with IC_50_ 33.7 ± 0.8 µg/mL, when compared to ascorbic acid, 14.2 ± 0.5 µg/mL. KA is used as a preservative and flavor enhancer because of its powerful antioxidizing and antimicrobial activities [70,71].

#### 2.3.3. Cytotoxic Activity

An increasing worldwide interest has been directed toward exploring new economic and safe anticancer drugs of natural origin, which might help in chemotherapy and/or chemoprevention of different types of cancer [72]. There has been a significant increase in the number of anticancer compounds isolated from endophytic fungi following the first report of the production of paclitaxel by a fungus [73]. An MTT assay was used to assess the cytotoxic impact on two tumor cell lines in the concentration range between 0 and 500 µg/mL.

**Figure 5:**
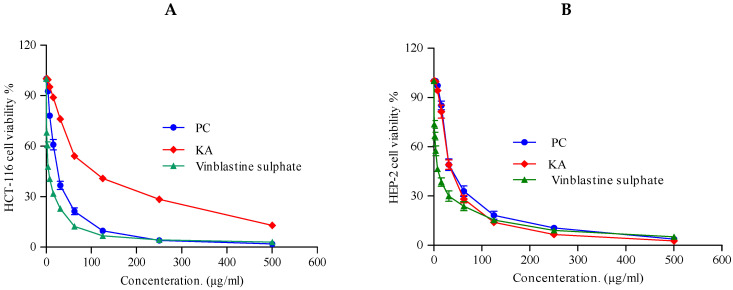
(**A**) Cytotoxic activity of *Penicillium chrysogenum* total extract (PC) and Kojic acid (KA). DMSO and vinblastine sulphate were used as negative and positive controls, respectively, against HCT-116 cell line at different concentrations. (**B**) Cytotoxic activity of *Penicillium chrysogenum* ethyl acetate extract (PC) and kojic acid (KA) against HEP-2 cell line at different concentrations. Data were analyzed by using one-way ANOVA and statistical significance was calculated with Dunnett’s multiple comparisons test. *p*-value < 0.05 was considered as statistically significant. The data display the mean ±SD of three biological replicas.

All tested fractions showed considerable cytotoxic activity against the cell lines under investigation (HCT-116 and HEP-2). In HCT-116 colon carcinoma cells (Figure 5A), both ethyl acetate extract of *P. chrysogenum* and KA showed strong cytotoxic activity with IC_50_ 22.6 ± 0.8 and 23.4 ± 1.4 µg/mL, respectively, as represented in Table 5. With respect to HEP-2 larynx carcinoma, as shown in Figure 5B, the total extract and KA possessed strong activity and similar cytotoxic profile against HEP-2 cell lines with equal IC_50_ 30.8 ± 1.3 and 30.8 ± 1.2 µg/mL, respectively, as shown in Table 5. These significant antimicrobial and cytotoxic activities might be attributed to the presence of penicillin G, KA, sohirnone B, and camptothecin as major constituents in the ethyl acetate extract of *P. chrysogenum*. It is noteworthy that penicillin G was used in the treatment of a variety of skin, urinary tract, and respiratory infections [74], and that both KA and camptothecin exhibit antibiotic and antineoplastic activities [75].

## 3. Materials and Methods

### 3.1. General Materials and Methods

The evaporation of solvents was achieved using a Buchi rotary evaporator (BUCHI R-100, Büchi Labortechnik AG, Flawil, Switzerland). A UV lamp λ_max_ 254/366 nm (Model UV GL-58, Upland, CA, USA) was used for thin layer chromatography (TLC) visualization. A circulating hot-air oven, WT-binder 7200 (BINDER GmbH, Tuttlingen, Germany), was used for heating and drying in this study. Infrared (IR) spectral analysis was recorded in potassium bromide disks on a Pye Unicam SP 3000 and an IR spectrophotometer (FT/IR-460 plus; JASCO International Co., Ltd., Easton, MD, USA).

An ultraperformance liquid chromatography-electrospray tandem mass spectrometry (UPLC-ESI-MS/MS) in negative and positive ionization modes were performed on a XEVO-TQD triple-quadruple instrument (Waters Corporation, Milford, MA, USA) mass spectrometer (ACQUITY UPLC BEH C18 (1.7 um, 2.1 × 50 mm) column; column flow rate 0.2 mL/min). The solvent system consisted of (A) water containing 0.1 percent formic acid and (B) methanol containing 0.1 percent formic acid (Ain Shams University, Cairo, Egypt).

Nuclear magnetic resonance (NMR) analysis 1D and 2D experiments were performed on a Bruker AMX 400 MHZ (Billerica, MA, USA) for ^1^HNMR and with standard pulse sequences operating at 100 MHz for ^13^CNMR. ^1^H-^13^C one-bond connectivity was detected with the HSQC gradient pulse factor selection. Two-bond and three-bond connectivity was identified by the HMBC experiment. Chemical shifts are reported in δ (ppm) and coupling constants (*J*) are reported in Hz. Unless otherwise mentioned, tetramethylsilane was the internal standard. In addition, DMSO_*d_6_* (solvent at room temperature) was of a spectroscopic grade for spectral analysis (Sigma-Aldrich, St. Louis, MO, USA). Furthermore, a pH meter (Hanna instruments 85/9, Haryana, India), Autoclave (Medexport BK-75, Frankfurt, Germany), and Laminar air flow cabinet (Jinan, Shandong, China) were also used.

### 3.2. Collection of Marine Sponge Samples, Isolation, and Morphological Identification of Endophytic Fungi

Marine sponge, namely, *Cliona* sp. and *Hymedesmia* sp. class *Demospongiae*, were collected from the Red Sea, specifically, 20 km away from Sharm el-Sheikh [27°45′57.8″ N 34° 22′10.8″ E], by scuba diving at a depth of 8:10 m off, during Nov–Dec/2018 Figure 6 (**1** and **2**). The collected material was immediately frozen and maintained at −20 °C until investigation, which was performed as soon as possible, to avoid the growth of ambient bacteria.

The sponge biomass was identified by Prof. Saad Zakaria, Marine Science Department, Faculty of Science, Suez Canal University. The sponges were brought to the lab in a sterile ice box, washed thoroughly with sterile sea water to eliminate adherent surface debris prior to isolation of their endophytic fungi, and then sectioned into small segments of approximately 1 cm × 1 cm. The parts were surface-sterilized with ethanol 70 percent (*v*/*v*) for 60–120 s, followed by 2.5 percent sodium hypochlorite for 2 min, and then rinsed with sterile sea water to avoid any epiphytic microbes [76,77].

**Figure 6:**
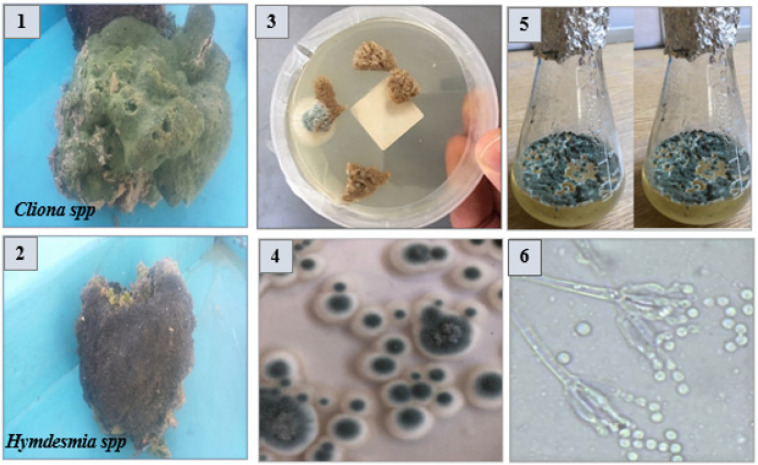
(**1** and **2**) The collected marine sponges (host organisms) *Cliona* sp. and *Hymedesmia* sp. (**3**) Isolation of endozoic fungi associated with marine sponge using malt agar and malt yeast agar media. (**4**) Purification of isolated strains on malt agar media. (**5**) Large scale fermentation on Wirkham’s media. (**6**) Identification of selected strain (*Penicillium chrysogenum*) according to the universal keys.

The surface-sterilized sponge parts were placed on the surface of malt yeast agar (MYA) and malt extract agar (MA) media [76,78] containing chloramphenicol (0.2 g), as shown in Figure 6 (**3**) and incubated for 15 days at 30 °C. Three biological replicates of each fungal isolate were conducted. The developed fungal hyphal tips were collected and purified by subculturing on the same media. Meanwhile, the purified fungal isolates were maintained as slope cultures at 4 °C until further screening analyses. The recovered fungal isolates were grown on MA media for 15 days at 30 °C and the developed colonies, as shown in Figure 6 (**4**,**6**), were examined every day based on their morphological features. Furthermore, they were identified on the basis of their species level according to the universal keys as colony diameter, extracellular exudates, pigmentation, mycelium color, conidial heads, fruiting bodies, and sporulation [15,16,17].

### 3.3. Preparation of P. chrysogenum Crude Ethyl Acetate Extract

The recovered *P. chrysogenum* fungal isolate from the selected medicinal marine sponge *Cliona* sp. and *Hymedesmia* sp. (two agar plugs of 5 mm each) of a six-day-old culture of malt agar medium [Bacto agar 15 g/L, malt extract 15 g/L, pH 7.4–7.8 (adjusted with NaOH/HCl), sea water ad 1000 g] was screened for its biological activity by growing it on Wickerham’s broth medium [yeast extract 3 g/L, malt extract 3 g/L, peptone 5 g/L, glucose 10 g/L, pH 7.2–7.4 (adjusted with NaOH/HCl), sea water ad 1000 g] for three weeks, as depicted in Figure 6 (**5**). An equal volume of ethyl acetate (300 mL) was added to each flask for 24 h. The flask content was added thoroughly into an Ultra Turrax (T18 basic, IKA) at 4000 μ min^−1^ for 10 min for cell destruction and extraction. The mixture was filtered, and the mycelium residue was discarded. The culture filtrate was transferred to a separating funnel and the ethyl acetate layer was isolated. The aqueous layer was extracted twice with 300 mL ethyl acetate. The combined ethyl acetate extract was washed with 100 mL demineralized water to eliminate any remaining sea salt and concentrated using a rotary evaporator at 50 °C to give (3 gm) solid residue.

### 3.4. Isolation of Kojic Acid from the Ethyl Acetate Extract of P. chrysogenum

Kojic acid was precipitated from the ethyl acetate extract of broth media after fermentation and concentration of the extract. The KA precipitate was washed several times with methanol to dispose of any impurities, redissolved in CH_2_Cl_2_, and recrystallized using acetone. It gave 168 mg of white needle crystals, with a melting point (m.p.) of 154 °C. It also gave a white spot with R_ƒ_ value 0.74 in solvent system CH_2_Cl_2_-MeOH (9.5 − 0.5) after spraying with vanillin or H_2_SO_4_ and heating. It also showed a protonated molecular ion peak at *m/z* 143.0706 [M + H]^+^ using UPLC-ESI-MS/MS (positive mode).

### 3.5. UPLC-ESI-MS/MS Profiling for Fungal Strain P. chrysogenum

Ultra-performance liquid chromatography with electrospray ionization quadrupole linear ion trap tandem mass spectrometry analysis was performed in the ESI-MS positive ion acquisition mode with a XEVO TQD triple quadruple instrument. The method, in a multiple-reaction monitoring (MRM) mode, was employed for the determination of phytochemicals. The ethyl acetate extract of the fungal strain *P. chrysogenum* was analyzed by UPLC to obtain the chemical profile. The samples were dissolved in HPLC grade methanol and then filtered through a 0.2 μm membrane disc filter. The resultant solution concentrations were in the range between 0.2 and 0.5 mg/mL, based on the crude extract. The Waters Corporation, Milford, MA 01757, USA mass spectrometer was the UPLC system.

Reverse-phase separations were performed [ACQUITY UPLC-BEH C 18 1.7 µm-2.1 × 50 mm Column; 50 mm × 1.2 mm (inner diameter) and 1.7 µm particle size] at a 0.2 m/mL flow rate. The gradient program applied for the analysis was previously used by [79]. The mobile phase consists of acidified water containing 0.1 percent formic acid (A) and acidified methanol containing 0.1 percent formic acid (B). The applied elution conditions were: 0–2 min 10 percent B isocratic; 2–5 min, linear gradient B 10 to 30 percent; 5–15 min, linear gradient from 30 percent to 70 percent B; 15–22 min, linear gradient from 70 percent to 90 percent B; 22–25 min, 90 percent B isocratic. Finally, the process included washing and reconditioning of column.

Electrospray Ionization (ESI) was carried out in a positive ion mode. The parameters for analysis were carried out using the positive ion mode as follows: source temperature 150 °C; cone voltage 30 eV; capillary voltage 3 kV; desolvation temperature 440 °C; cone gas flow 50 L/h; and desolvation gas flow 900 L/h. Mass spectra were detected in the ESI between m/z 100–1000 atomic mass units. The chemical constituents were identified by their ESI-QqQLIT–MS/MS spectra and fragmentation patterns. The peaks and spectra were processed using the Maslynx 4.1 software(Waters Corporation, Milford, MA, USA) and tentatively identified by comparing the retention time (Rt) and mass fragmentation with the reported data. Most analytes were detected as single-charged ions, specifically, either as protonated [M + H]^+^ or sodium adducts [M + Na]^+^, in positive mode.

### 3.6. Antioxidant Activity

The antioxidant activity of the extract was determined at the Regional Center for Mycology and Biotechnology (RCMB) at Al-Azhar University by the DPPH free radical scavenging assay, wherein triplicate and average values were considered.

#### DPPH Radical Scavenging Activity

A freshly prepared (0.1 mM) solution of 2,2-diphenyl-1-picrylhydrazyl (DPPH) and different tested extracts prepared at 5, 10, 20, 40, 80, 160, and 320 µg/mL in methanol were vigorously mixed and allowed to stand for 30 min at room temperature in the dark [80]. The absorbances of the resultant solution were recorded with a UV-visible spectrophotometer (Milton Roy, Spectronic 1201, Houston, TX, USA) at λ_max_ 517 nm. A DPPH radical in methanol with the reference antioxidant compound (ascorbic acid), or without the antioxidant sample, was used as positive and negative controls. The reference compound, ascorbic acid, was used at different concentrations (5, 10, 20, 40, 80, 160, and 320 µg/mL). All the determinations were performed in three replicates and averaged. The percentage inhibition of the DPPH radical was calculated according to the following formula:

PI = [{(*A*C − *A*S)/*A*C} × 100]

where PI is the percentage inhibition of the DPPH radical, AC is the absorbance of the control solution, and AS is the absorbance of the sample in DPPH solution. The percentage of DPPH radical scavenging was plotted against each extract concentration and ascorbic acid (µg/mL) to determine scavenging capacity (IC_50_), which is the concentration required to scavenge DPPH by 50 percent (that is, the concentration facilitating 50 percent reduction in the absorbance of a DPPH solution from its initial absorbance).

### 3.7. Cytotoxic Activity

The cytotoxic impact of the ethyl acetate extract of *P. chrysogenum* on HEP-2 (Human larynx carcinoma) and HCT-116 (Colon carcinoma) was investigated using 3-(4, 5-dimethylthiazole-2-yl)-2, 5-diphenyl-tetrazolium bromide (MTT) against DMSO and vinblastine sulphate as negative and positive controls, respectively. HEP-2 and HCT-116 cell lines were obtained from the VACSERA Tissue Culture Unit. Dimethyl sulfoxide (DMSO), crystal violet, and trypan blue dye were purchased from Sigma (St. Louis, MO, USA). Fetal Bovine serum, DMEM, RPMI-1640, HEPES buffer solution, L-glutamine, gentamycin, and 0.25% Trypsin-EDTA were purchased from Lonza (Basel, Switzerland). The crystal violet stain (1 percent) is composed of 0.5 percent (*w*/*v*) crystal violet and 50 percent methanol, then made up to volume with ddH_2_O, and filtered through a Whatmann No. 1 filter paper. The cells were propagated on Dulbecco’s modified Eagle’s medium (DMEM), supplemented with 10 percent heat-inactivated fetal bovine serum, 1 percent L-glutamine, HEPES buffer, and 50 µg/mL gentamycin. The cells were maintained at 37 °C in a humidified atmosphere with 5 percent CO_2_, and they were sub-cultured two to three times a week.

In the case of antitumor assays, the cells were seeded in 96-well plate at a cell concentration of 1 × 10^4^ cells per well in 100 µL of growth medium. Fresh medium containing different concentrations of the test compound and the extract were added after 24 h of seeding. Serial two-fold dilutions of the tested compound and the extract were added to confluent cell monolayers dispensed into 96-well, flat-bottomed microtiter plates (Falcon, NJ, USA) using a multichannel pipette. The microtiter plates were incubated at 37 °C in a humidified incubator with 5 percent CO_2_ for a period of 24 h. Three wells were used for each concentration of the test sample. Control cells were incubated without the test sample and with or without DMSO. The small percentage of DMSO present in the wells (maximal 0.1 percent) was found to not affect the experiment. After incubation of the cells at 37 °C for 24 h, the viable cells’ yield was determined by a colorimetric method.

Briefly, after the end of the incubation period, media were aspirated, and the crystal violet solution (1 percent) was added to each well for at least 30 min. The stain was removed, and the plates were rinsed using tap water until all excess stains were removed. Glacial acetic acid (30 percent) was then added to all wells and mixed thoroughly. Subsequently, the absorbance of the plates was measured after gently shaking the same on a microplate reader (TECAN, Inc., Morrisville, NC, USA), using a test wavelength of 490 nm. All results were corrected for background absorbance detected in wells without added stains. The treated samples were compared with the cell control in the absence of the tested compounds. All experiments were carried out in triplicate. The cell cytotoxic effect of each tested compound was calculated.

The optical density was measured with the microplate reader (SunRise, TECAN, Inc., Morrisville, NC, USA) to determine the number of viable cells, and the percentage of viability was calculated, as the tumor cell lines were suspended in medium at a concentration of 5 × 10^4^ cell/well in Corning^®^ 96-well tissue culture plates, and then incubated for 24 h. The tested extracts were then added into 96-well plates (six replicates) to achieve eight concentrations for each extract. Six vehicle controls with media or 0.5 percent DMSO were run for each 96-well plate as a control. After incubating for 24 h, the numbers of viable cells were determined by the MTT test.

Cell viability percentage = [1 − (OD_t_/OD_c_)] × 100 percent, where OD_t_ is the mean optical density of wells treated with the tested sample, and ODc is the mean optical density of untreated cells. The relation between surviving cells and each extract concentration (1–500 µg/mL) is plotted to find the survival curve of each tumor cell line after treatment with the tested fraction. The 50 percent inhibitory concentration (IC_50_), the concentration required to cause toxic effects in 50 percent of intact cells, was estimated from graphic plots of the dose response curve for each concentration using non-linear regression analysis of GraphPad Prism 5 software (GraphPad Software, San Diego, CA, USA) [81,82]. All experiments were repeated at least three times. Results are reported as mean ± SD.

### 3.8. Antimicrobial Activity (Well Diffusion Method)

The antibacterial activity of *P. chrysogenum* ethyl acetate extract and kojic was evaluated by using the well diffusion method [83] against *Staphylococcus aureus* (*S. aureus*, ATCC 5368) as gram-positive bacteria. *Escherichia coli* (*E. coli*, ATCC 10536) and *Pseudomonas aeruginosa* *(P. aeruginosa*, ATCC 27853) as gram-negative bacteria, along with *Candida albicans* (*C. albicans,* ATCC 10231), *Aspergillus fumigatus* (*A. fumigatus*, RCMB 002008), *Aspergillus flavus* (*A. flavus* RCMB 002002), *Cryptococcus neoformans* (*C. neoformans* RCMB 0049001), and *Fusarium oxysporum* (*F. oxysporum* RCMB 001004) as fungi. All the tested strains were obtained from the Regional Center for Mycology and Biotechnology (RCMB) at Al-Azhar University, Egypt. The assay was performed on Muller Hinton Agar (MHA) medium for bacterial strains and potato dextrose agar for fungi. The tested samples were dissolved in dimethyl sulfoxide (DMSO) at a concentration of 500 µg/mL. Ciprofloxacin (100 µg/mL) was used as a standard antibacterial drug, while Fluconazole (100 µg/mL) was used as a positive control for fungi. The wells were filled with 100 μL from a stock solution of each sample, with the standards and DMSO as a negative control. Cultures were incubated at 37 °C for 14–18 h for bacteria, and for 24–48 h for fungi. All the assays were conducted in triplicate. The activity was determined by measuring the diameter of the inhibition zone (mm) from three independent experiments, and the values were averaged. [84,85].

#### Determination of Minimum Inhibitory Concentration (MIC)

The MIC values of the tested compounds were determined using the agar dilution method. The tested compounds were dissolved and serially diluted in dimethyl sulfoxide (DMSO). A series of agar plates containing the assigned serial dilution of each compound was prepared. Each plate was prepared by mixing the indicated volume of the dissolved compound with molten Mueller–Hinton agar (MHA) medium to obtain agar with thickness between 3 and 4 mm, with final concentrations of DMSO not exceeding 5 percent and 2.5 percent for bacteria and fungi, respectively. The tested bacterial strains were grown overnight on MHA, and purified colonies were suspended in 0.9 percent saline. The turbidity of the bacterial inoculum was adjusted to equal 0.5 McFarland (2.5 × 10^8^ cfu/mL) and then diluted to one-tenth of the solution with saline. Prepared MHA plates were inoculated by delivering 2 µL of the prepared inoculum on their surfaces to obtain a final concentration of 10^4^ cfu per spot [86]. The tested fungal strains were streaked on potato dextrose agar, and purified colonies were suspended in saline. The turbidity of the prepared inoculum was adjusted to be equal to the turbidity of 0.5 Mcfarland (5 × 10^6^ cfu/mL), then diluted to 1:10 with saline. Prepared potato dextrose agar containing the serially prepared concentration of each compound was inoculated by delivering 2 µL of the prepared inoculum, so the final concentration of the inoculum was 10^3^ per the resultant spot. Inoculated plates were incubated at 30 °C for 24–48 h and were examined for the presence of fungal growth [87,88].

### 3.9. Fungal Deposition

The Internal Transcribed Spacer (ITS) sequence of the isolate *Penicillium chrysogenum* EFBL, an endozoic of *Cliona* sp., was deposited to the GenBank with accession No. OL597937.1.

### 3.10. Statistical Analysis

The data were collected and plotted using GraphPad Prism 5 software (GraphPad Software, San Diego, CA, USA). The data were analyzed by using one-way ANOVA and statistical significance was calculated with Dunnett’s multiple comparisons test. A *p*-value < 0.05 was considered statistically significant. The data display the mean ± SD of three biological replicas.

## 4. Conclusions

In the present study, *Penicillium chrysogenum*, an endozoic fungus recovered from *Cliona* sp. marine sponge, was investigated for the first time. The UPLC-ESI-MS/MS analysis revealed the tentative identification of 36 compounds, including amphetamine, pyrroline carboxylic acid, 3-hydroxy KA, KA, penicillin G, camptothecin, dihydrosorbicillin, sohirnone B, kynurenine, quinolactiocide, and sorrentanone as major compounds. KA was also identified from *Penicillium chrysogenum* ethyl acetate extract, and its structure was confirmed by IR, ESI-MS/MS, 1D and 2D NMR spectroscopy. The antioxidant, cytotoxic, and antimicrobial activities of the *P. chrysogenum* extract and KA were also investigated. Both the extract and KA showed significant cytotoxic activity against HCT-116 and HEP-2 cell lines. However, only KA showed a potent antioxidant activity compared to the *P. chrysogenum* extract, which is nearly inactive. Additionally, the PC extract and KA showed potent antibacterial activity against *S. aureus* and *P. aeruginosa*. Interestingly, the *P. chrysogenum* extract showed significant antifungal activity against *C. albicans*, *C. neoformans*, and *F. oxysporum*. In conclusion, based on the previous results, *Penicillium chrysogenum* isolated from *Cliona* sp. is a promising natural source of potent biologically active compounds for multi-drug resistant infections. Further studies of such endozoic fungi may result in the isolation of new natural drugs with variable therapeutic activities.

## Figures and Tables

**Table 1:** Screening for antimicrobial activity of endophytic fungi inhabiting the tested medicinal sponge.

Fungal Isolates			Gram Positive Bacteria		Gram Negative Bacteria				Fungi	
			*Staphylococcus aureus* ATCC 5368		*Escherichia coli* ATCC 10536		*Pseudomonas aeruginosa* ATCC 27853		*Candida albicans* ATCC 10231	
			IZ	MIC	IZ	MIC	IZ	MIC	IZ	MIC
*Cliona* sp.	1	*Aspergillus orchaceous* (C2)	28	2000	-	-	-	-	14	>3000
	2	*Aspergillus terreus* (C4)	14	1000	-	-	-	-	-	-
	3	*Aspergillus niger* (C5)	-	-	-	-	-	-	14 (>3000)	>3000
	4	*Penicillium Chrysogenum* (C6)	23	250	-	-	-	-	40	93.75
*Hymedesmia* sp.	1	*Aspergillus terreus* (H2)	19	250	-	-	-		20	187.5
	2	*Aspergillus awamori* (H3)	-	-	-	-	-	-	-	-
	3	*Aspergillus niger* (H4)	-	-	-	-	-	-	-	-
	4	*Aspergillus oryzae* (H5)	14	>2000	14	>2000	25	250	25	750
	5	*Alternaria alternata* (H6)	20	>2000	15	>2000	23	>2000	25	>3000
	9	*Trichoderma viridae* (H7)	21	>2000	14	>2000	-	-	18	>1500
	7	*Penicillium lilacinum* (H8)	-	-	-	-	-	-	14	>3000
	8	*Aspergillus astus* (H9)	-	-	-	-	-	-	-	-
	Ciprofloxacin		-	1.56 ± 1.2	-	3.125 ± 0.89	-	3.125 ± 0.24	-	-
	Fluconazole		-	-	-	-	-	-	42 ± 0.58	50 ± 0.24
	DMSO (Negative control)		10	-	10	-	19	-	12	-

IZ: Inhibition zone (mm) diameter; MIC: Minimum Inhibitory Concentration (μg/mL). MIC: 50–500 μg/mL (strong activity), 600–1500 μg/mL (moderate activity), >1500 μg/mL (weak activity) [18,19].

**Table 3:** Antibacterial activity of *Penicillium chrysogenum* (PC) total extract and kojic acid (KA) by agar diffusion method.

Extract/Compound	Inhibition Zone (IZ mm) Diameter (Mean ± SD)/Minimum Inhibitory Concentration (MIC µg/mL)					
	Gram Positive Bacteria		Gram Negative Bacteria			
	*Staphylococcus aureus* ATCC 5368		*Escherichia coli* ATCC 10536		*Staphylococcus aureus* ATCC 5368	
	IZ	MIC	IZ	MIC	IZ	MIC
Solvent (DMSO)	10		10		19	
PC Total extract	23 ± 0.72	250 ± 0.82	-	-	-	-
Kojic acid (KA)	14 ± 0.82	>2000 ± 1.4	14 ± 0.59	>2000 ± 1.5	25 ±0.82	250 ± 0.82
Ciprofloxacin	-	1.56 ± 1.2	-	3.125 ± 0.89	-	3.125 ± 0.24

MIC: 50–500 μg/mL (strong activity), 600–1500 μg/mL (moderate activity), >1500 μg/mL (weak activity) [18,19].

**Table 4:** Antifungal activity of *Penicillium chrysogenum* (PC) total extract and kojic acid (KA) by agar diffusion method.

Extract/ Compound	Inhibition Zone (IZ mm) Diameter (Mean ± SD)/Minimum Inhibitory Concentration (MIC µg/mL)									
	Fungi									
	*Candida albicans* ATCC 10231		*Aspergillus fumigatus* RCMB 002008		*Aspergillus flavus* RCMB 002002		*Cryptococcus neoformans* RCMB 0049001		*Fusarium oxysporum* RCMB 001004	
	IZ	MIC	IZ	MIC	IZ	MIC	IZ	MIC	IZ	MIC
PC Total extract	40 ± 0.45	93.75 ± 0.55	13 ± 0.63	625 ± 1.3	10 ± 0.73	1000 ± 1.3	22 ± 0.19	19.53 ± 0.48	9 ± 0.68	10000 ± 1.5
Kojic acid (KA)	25 ± 0.56	750 ± 0.38	13 ± 0.48	312.5 ± 0.47	9 ± 0.72	5000 ± 1.4	18 ± 0.58	39.06 ± 0.98	19 ± 0.93	39.06 ± 0.85
Fluconazole	42 ± 0.58	50 ± 0.24	18 ± 1.2	39.06 ± 0.72	17 ± 0.8	39.06 ± 0.48	25 ± 0.63	4.88 ± 0.32	19 ± 0.7	19.53 ± 0.82

MIC: 50–500 μg/mL (strong activity), 600–1500 μg/mL (moderate activity), >1500 μg/mL (weak activity) [18,19].

**Table 5:** Half maximum inhibitory concentration (IC_50_) of *Penicillium chrysogenum* (PC) total extract and kojic acid (KA) in cell viability of HCT-116 and HEP-2 cells after treatment for 48 h, as measured by the MTT assay. The data are presented as µg/mL.

Cell Line	Tested Fractions		
	IC_50_ (µg/mL)		
	PC	KA	Vinblastine Sulphate
HCT-116 (Colon carcinoma)	22.6 ± 0.8	23.4 ± 1.4	2.34 ± 0.28
HEP-2 (Human Larynx carcinoma)	30.8 ± 1.3	30.8 ± 1.2	6.61 ± 0.59

PC, *Penicillium chrysogenum* total extract; KA, kojic acid. The assay was conducted in triplicate.

## Data Availability

Not applicable.

## Supplementary Materials

The following supporting information can be downloaded at: https://www.mdpi.com/article/10.3390/md20050326/s1, Table S1. ^1^HNMR (400 MHz, DMSO_*d_6_*) and ^13^C NMR (100 MHz, DMSO_*d_6_*) data for kojic acid, Figure S1. Positive ESI-MS/MS spectrum of kojic acid, Figure S2. IR Spectrum of kojic acid, Figure S3. ^1^HNMR spectrum of kojic acid in DMSO_d_6_, Figure S4. ^13^C NMR spectrum of compound kojic acid in DMSO_d_6_, Figure S5. ^1^H-^13^C HSQC spectrum of kojic acid, Figure S6. ^1^H-^13^C HMBC spectrum of kojic acid, Figure S7. HMBC correlations of kojic acid.
