# Supplementary material for: Metabolic Profiling and In Vitro Assessment of the Biological Activities of the Ethyl Acetate Extract of Penicillium chrysogenum “Endozoic of Cliona sp. Marine Sponge” from the Red Sea (Egypt)
Source: Mar Drugs. 2022 May 15;20(5):326. doi: 10.3390/md20050326 (PMC9143181; doi:10.3390/md20050326)
Supplement: Supplementary file 1 [file marinedrugs-20-00326-s001.zip › marinedrugs-1727980-SI.pdf]

Supplementary Materials

Metabolic Profiling and *In Vitro* Assessment of the Biological Activities of the Ethyl Acetate Extract of *Penicillium chrysogenum* "endozoic of *Cliona spp* Marine Sponge," from the Red Sea (Egypt)

Muneera S. M. Al-Saleem[1], Wafaa H. B. Hassan[2], Zeinab I. El Sayed[2], Mahmoud M. Abdel-Aal[2], Wael M. Abdel-Mageed[3, 4*] Eman Abdelsalam[2], and Sahar Abdelaziz[2*]

[1] Department of Chemistry, Science College, Princess Nourah Bint Abdulrahman University, Riyadh 11671, Saudi Arabia; msalsaleem@pnu.edu.sa.

[2] Department of Pharmacognosy, Faculty of Pharmacy, Zagazig University, 44519 Zagazig, Egypt; wafaahbh@zu.edu.eg (W. H. B. H); zainb_ebrahim@zu.edu.eg (Z. I. E); Mahmoud.ibrahim@su.edu.eg (M. M. A) eaamer@zu.edu.eg (E. A)

[3] Department of Pharmacognosy, College of Pharmacy, King Saud University, P.O. Box 2457, Riyadh 11451, Saudi Arabia; wabdelmageed@ksu.edu.sa.

[4] Department of Pharmacognosy, Faculty of Pharmacy, Assiut University, Assiut 71526, Egypt. waelcognosy@yahoo.com.

* Correspondence: wabdelmageed@ksu.edu.sa (W. M. A. M); sah_abdelaziz@zu.edu.eg (S. A)

List of content

- **Table S1.** ^{1}HNMR (400 MHz, DMSO_d_6) and ^{13}C NMR (100 MHz, DMSO_d_6) data for kojic acid.
- **Figure S1.** Positive ESI-MS/MS spectrum of kojic acid.
- **Figure S2.** IR Spectrum of kojic acid.
- **Figure S3.** ^{1}HNMR spectrum of kojic acid in DMSO_d_6.
- **Figure S4**. ^{13}C NMR spectrum of compound kojic acid in DMSO_d_6.
- **Figure S5.** HSQC spectrum of kojic acid.
- **Figure S6.** HMBC spectrum of kojic acid.
- **Figure S7.** HMBC correlations of kojic acid.

Table S1. ^{1}H NMR (400 MHz, DMSO_*d*$_6$) and ^{13}C NMR (100 MHz, DMSO_*d*$_6$) data for kojic acid

Position	δc, ppm (mult.)	δH, ppm [mult., *J*(HZ)]	HMBC (C→ H)
2	168	--	
3	109.84 (d)	6.33 (s)	H_7, H_8
4	173.9 (s)	--	
5	139.3 (s)	--	
6	145.7 (d)	8.02 (s)	H_3, H_6
7	59.5 (t)	4.29 (s)	H_3, H_8
8	--	5.65 (t)	
9	--	9.04 (s)	

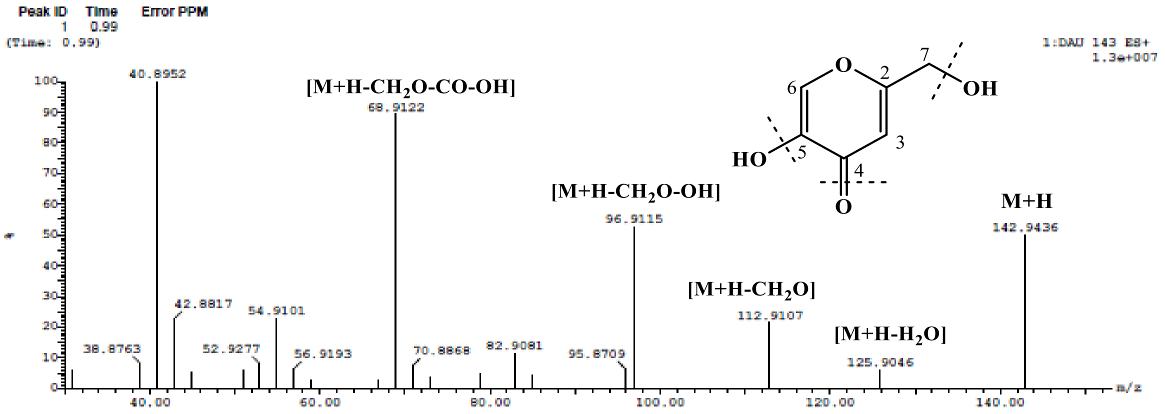

Figure S1. Positive ESI-MS/MS spectrum of kojic acid

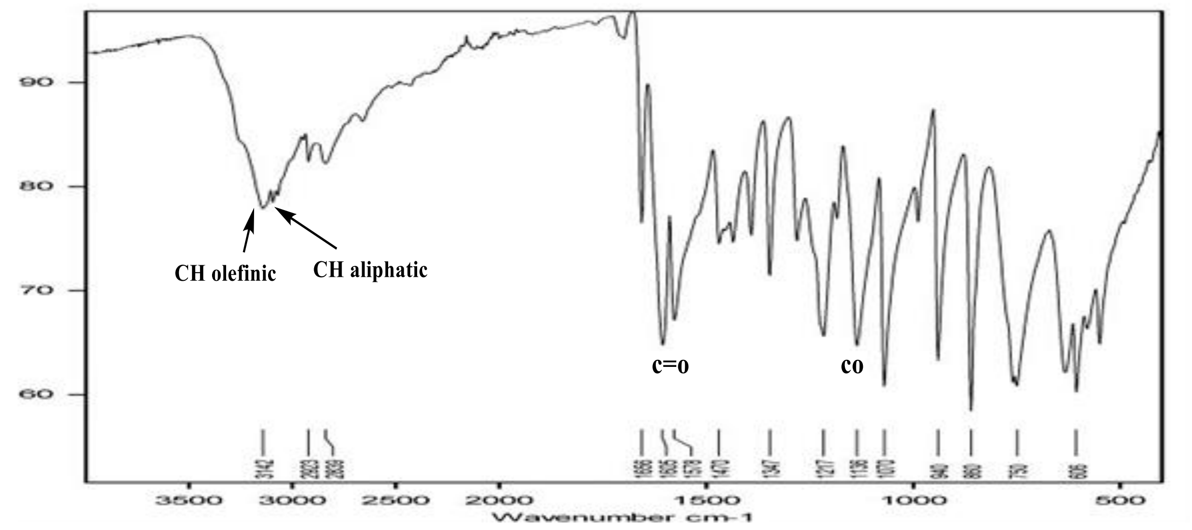

Figure S2. IR spectrum of kojic acid

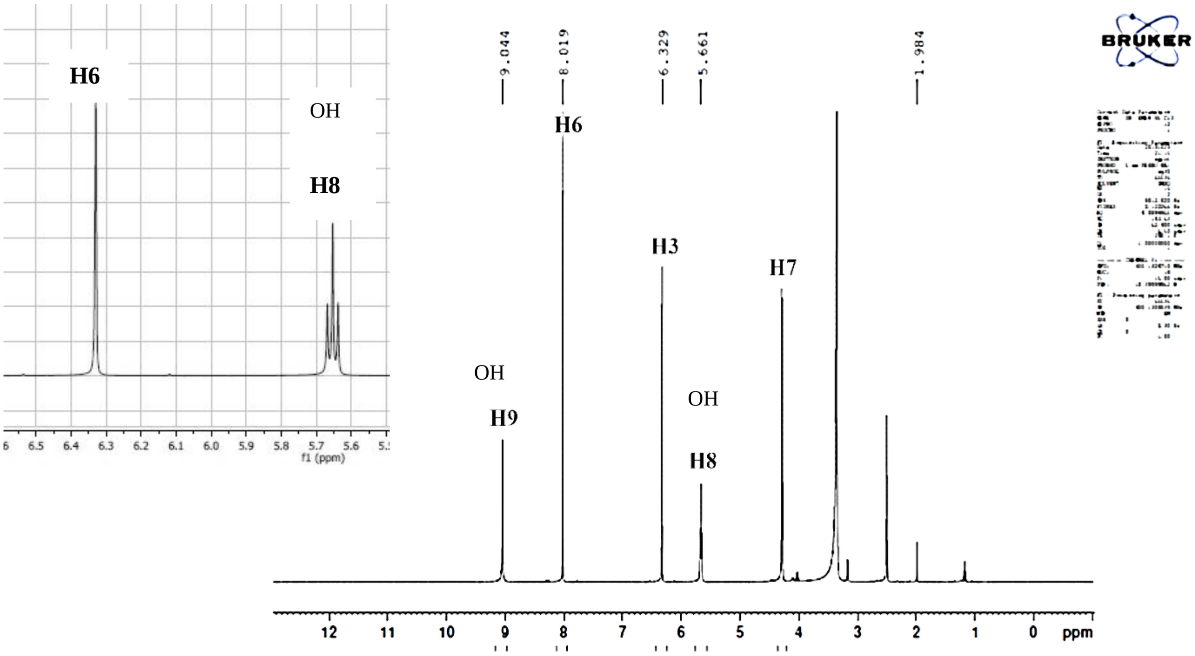

Figure S3. ^{1}H NMR spectrum of kojic acid

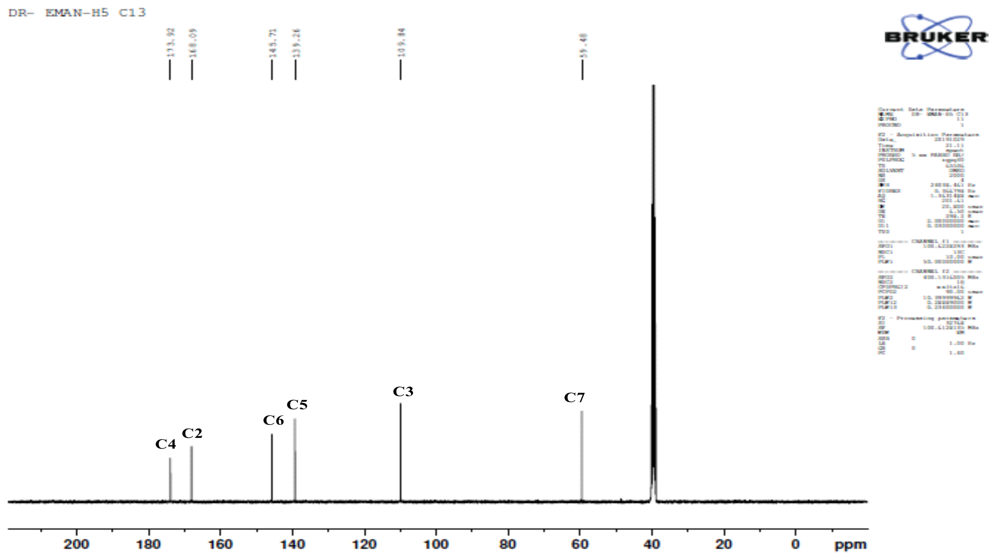

Figure S4. ^{13}C spectrum of kojic acid

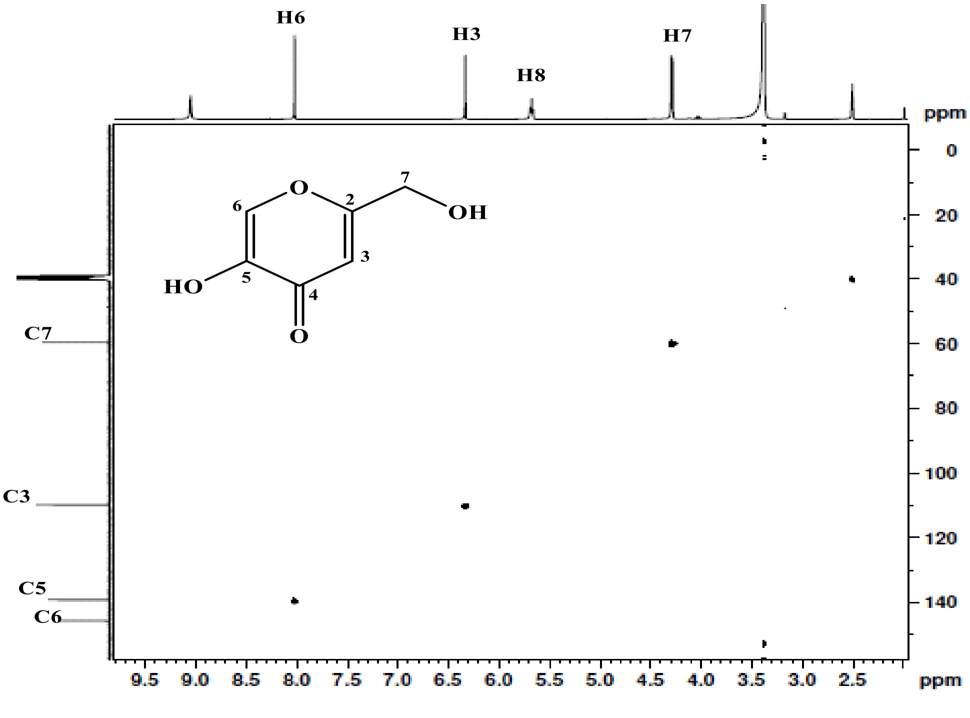

Figure S5. HSQC spectrum of kojic acid

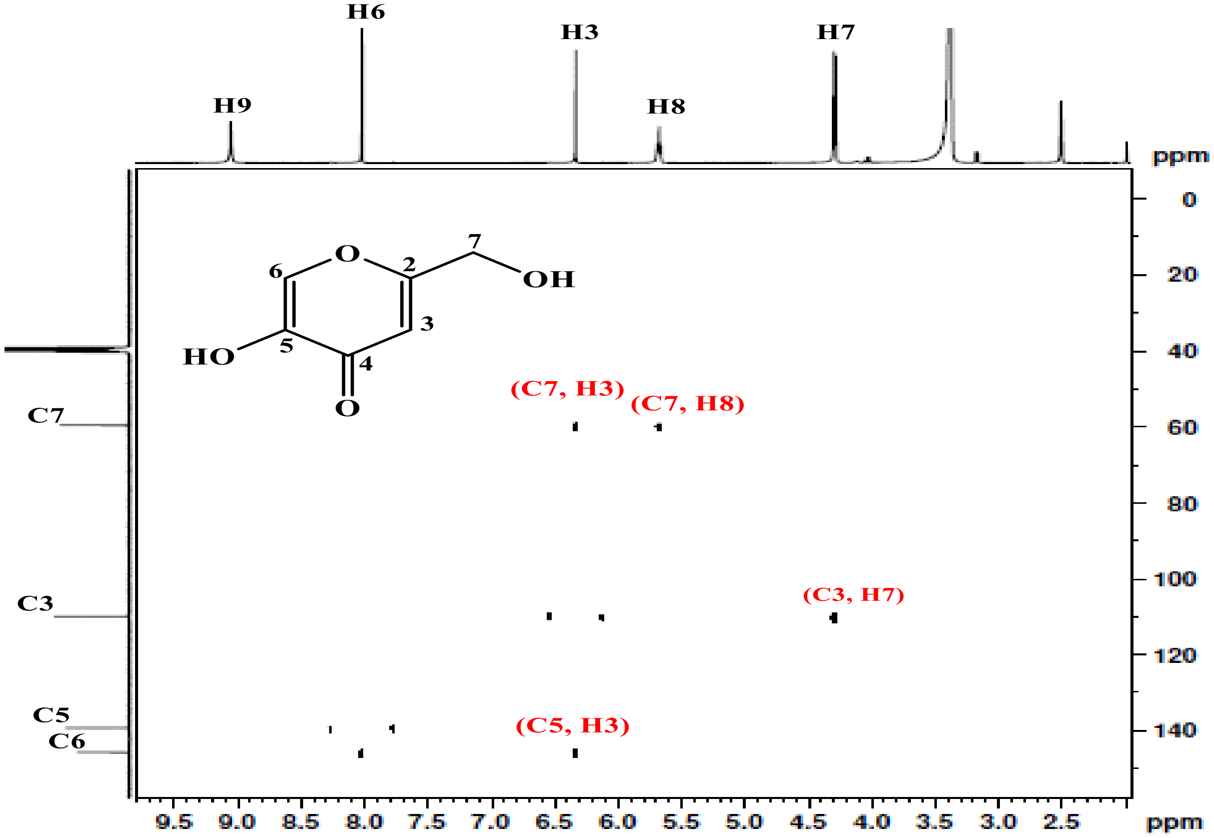

Figure S6. HMBC spectrum of kojic acid

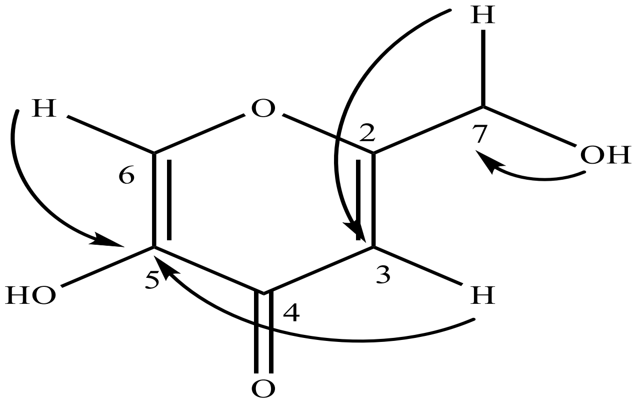

Figure S7. HMBC correlation of kojic acid
